# Prefrontal Cortical to Mediodorsal Thalamus Projection Neurons Regulate Posterror Adaptive Control of Behavior

**DOI:** 10.1523/ENEURO.0254-22.2022

**Published:** 2022-11-02

**Authors:** Bastiaan Bruinsma, Tommy Pattij, Huibert D. Mansvelder

**Affiliations:** 1 Department of Integrative Neurophysiology, Center for Neurogenomics and Cogntive Research (CNCR), Vrije Universiteit Amsterdam, Amsterdam 1081 HV, The Netherlands; 2 Brain Research & Innovation Centre, Ministry of Defence, Utrecht 3584 EZ, the Netherlands; 3 Department of Anatomy and Neurosciences, Amsterdam University Medical Centers, Vrije Universiteit Medical Center, Amsterdam 1081 HZ, The Netherlands

**Keywords:** 5-CSRTT, adaptive control, cognitive control, mediodorsal thalamus, prefrontal cortex

## Abstract

Adaptive control is the online adjustment of behavior to guide and optimize responses after errors or conflict. The neural circuits involved in monitoring and adapting behavioral performance following error are poorly understood. The prefrontal cortex (PFC) plays a critical role in this form of control. However, these brain areas are densely connected with many other regions, and it is unknown which projections are critical for adaptive behavior. Here, we tested the involvement of four distinct dorsal and ventral prefrontal cortical projections to striatal and thalamic target areas in adaptive control. We re-analyzed data from published experiments, using trial-by-trial analyses of behavior in an operant task for attention and impulsivity. We find that male rats slow their responses and perform worse following errors. Moreover, by combining retrograde labeling and chemogenetic silencing, we find that dorsomedial prefrontal pyramidal neurons that project to the lateral nucleus of the mediodorsal thalamus (MDL) are involved in posterror performance and timing of responses, specifically with unpredictable delays until stimulus presentation. Together, these data show that dorsal medial PFC (mPFC) projection neurons targeting the lateral MDT regulate adaptive control to flexibly optimize behavioral responses in goal-directed behavior.

## Significance Statement

Adaptive control is the online adjustment of behavior to guide and optimize responses after errors or conflict. Whereas this type of behavioral control is affected in several mental disorders, the brain mechanisms involved are incompletely understood. Here, we use trial-by-trial analysis of behavior in an operant task for attention and impulsivity, in rats. Since we know the prefrontal cortex (PFC) plays a critical role in this type of behavior, we chemogenetically silenced four populations of cells in the PFC that each project to a different subcortical region. We find that one population, projecting to the lateral nucleus of the mediodorsal thalamus (MDL), is involved in this flexible type of behavior.

## Introduction

In goal-directed behavior, cognitive control mechanisms are important to select the appropriate set of behaviors while suppressing reflexive and impulsive behaviors as well as distracting stimuli. Following failures to acquire a particular goal, individuals subsequently adjust their behavioral responses to improve success, which is guided by increased cognitive control (Ridderinkhof et al., 2004). For instance, individuals with schizophrenia exhibit deficits in cognitive control (Guo et al., 2019), as well as impairments in adjusting behavior after errors or conflicts (Kerns et al., 2005).

Rodents also adapt behavioral performance and slow their responses after error trials (Narayanan et al., 2013; De Haan et al., 2018). Pyramidal neurons in the dorsal part of the rodent medial prefrontal cortex (mPFC) increase activity after error responses and show persistent increased activity throughout the subsequent trial (Totah et al., 2009; Pinto and Dan, 2015). In line with this, pharmacological inhibition of the mPFC abolishes the posterror slowing of responses (Narayanan et al., 2013). However, the mPFC contains pyramidal cells that project to various cortical and subcortical targets (Gabbott et al., 2005). In view of this heterogeneity, it is poorly understood which projection neuron populations are involved in adaptive control. Recently, optogenetic stimulation of anterior cingulate cortex (ACC) neurons projecting to visual cortex was shown to improve attentional performance in trials following errors in mice (Norman et al., 2021).

Recently, we found that mPFC projection neurons exert circuit specific effects on cognitive control in the self-paced five-choice serial reaction time task (SP-5-CSRTT), based on their projection target. Specifically, inhibiting a neuronal population that projects to the lateral nucleus of the mediodorsal thalamus (MDL) decreased impulsive (premature) responding, whereas inhibition of projection neurons to the medial nucleus of the thalamus (MDM) increases impulsive responding. Additionally, we showed that inhibition of neurons that project to the dorsomedial striatum (DMS), but not the ventromedial (VMS) striatum, leads to an increase in impulsive responses (de Kloet et al., 2021). It is unknown whether any of these corticothalamic or corticostriatal projections are involved in adaptive control of behavior.

Projections from the mPFC to the nuclei of the mediodorsal thalamus (MDT) or striatum are likely involved in this adaptive control of behavior. The MDT plays a role in behavioral flexibility (Parnaudeau et al., 2015; Marton et al., 2018) and the MDT achieves this by modulation of mPFC activity via recurrent projections (Rikhye et al., 2018). The striatum is also involved in adaptive control. In the DMS, dopamine receptor type-2 (D2R)-expressing neurons signaled when no reward was earned in the current trial and encoded the switch of behavioral strategy in the next trial (Nonomura et al., 2018). During risky decision-making, D2R-expressing neurons in the VMS showed activity that signaled loss and predicted upcoming safe behavioral choices (Zalocusky et al., 2016). Taken together, these findings clearly indicate that both the prefrontal cortex and several of its output targets are involved in adaptive control of behavior. This idea is supported by a recent study showing that mPFC neurons projecting to the MDT and VMS maintain representations of recent trial outcomes and feedback (Spellman et al., 2021).

In the current study, we aimed to investigate whether medial prefrontal projection populations that project to the MDL, MDM, DMS, and VMS are involved in adaptive control of behavior. For this, we re-analyzed data from our previous publication (de Kloet et al., 2021) using a trial-by-trial analysis. We used the self-paced five-choice serial reaction time task (SP-5-CSRTT), a home-cage based, automated variant of a task to measure visuospatial attention and impulsivity (Robbins, 2002). We found that animals show adaptive control after error responses and difficult trials. Next, we analyzed data from viral retrograde labeling methods combined with chemogenetic inhibition in the SP-5-CSRTT and found that specifically dorsal mPFC to MDL projection neurons play a role in this behavior.

## Materials and Methods

### Animals

For training and testing in CombiCages (Bruinsma et al., 2019); 84 male Long–Evans rats (eight weeks old) were initially housed in pairs with food and water available *ad libitum* one to two weeks before surgeries. Because of vendor-related issues, rats in the cortico-thalamic chemogenetic experiments were ordered from Charles River (Den Bosch), whereas rats in the trial-by-trial analysis and cortico-striatal inhibition experiment were ordered from Janvier. Importantly, both group of rats had their own respective control group. See Extended Data Figure 1-1 for the study design. The same animals were used to test inhibition of mPFC projection neurons on SP-5-SCRTT behavior (de Kloet et al., 2021), and we here analyzed the behavioral data to test the role of mPFC projection neurons in behavioral adaptation. After recovery from surgery and habituation, animals were housed individually in CombiCages under a 12/12 h light/dark cycle (lights off at 12 P.M.) and behavioral procedures were initiated as described previously (Bruinsma et al., 2019). All experimental procedures were in accordance with European and Dutch law and approved by the animal ethical care committee of the VU University and VU University Medical Center.

### Viral vectors

We infused the retrograde virus CAV-2-Cre (IGMM) in the MDL/MDM (0.345 μl, 5 × 10^12^ particles/ml) to label cortico-thalamic projection neurons or in the DMS/VMS (0.483 μl, 1.25 × 10^12^ particles/ml) to label cortico-striatal projection neurons. Subsequently, we expressed the DREADD-receptor hM4D(Gi) in the mPFC using AAV5-+EF1α-DIO-hm4D(Gi)-mCherry (UZH, 0.483 μl, 3.6 × 10^12^ particles/ml). Control animals were injected with DIO-eYFP (0.483 μl, 4.2 × 10^12^ particles/ml). Titers and infusion volumes of the different viral vectors were determined in pilot experiments and the same as reported previously (Terra et al., 2020; de Kloet et al., 2021).

### Surgery

Rats were anaesthetized with 2.5% isoflurane in a mixture of air and oxygen (1.2 l/min). The animals were placed on a heating pad in a stereotaxic apparatus (Kopf). The skull was exposed by retracting the skin of the scalp. We used a Nanoject II injector (Drummond Scientific) to infuse virus under a 10° angle in the following regions: DMS: anteroposterior (AP): +1.44 mm; mediolateral (ML): ±2.78 mm, dorsoventral (DV): −4.47 mm. VMS: (AP +1.44, ML ±2.59, DV 7.41 and 6.8). MDL: (AP −3, ML ±2.32, DV 5.89). MDM: (AP −3, ML 1.42, DV 5.89). Dorsal mPFC: (AP +2.76, ML ±1.3, DV −2.9). Ventral mPFC: (AP +2.76, ML ±1.47, DV −4.87). All infusions were performed bilaterally and after the surgery the animals received an additional injection with 1 ml 0.9% saline to rehydrate. Rimadyl (carprofen 5 mg/kg) and Temgesic (buprenorphine, 0.05 mg/kg) were used as postoperative analgesics. Rimadyl was additionally administered 1 d before and until 2 d after the surgery. Lidocaine (xylocaine) was used as local anesthetic of the scalp during surgery.

### SP-5-CSRTT task

For an elaborate description of the automated self-paced version of the five-choice serial reaction time task (SP-5-CSRTT), see Remmelink et al. (2017) and Bruinsma et al. (2019).

**Figure 1.**
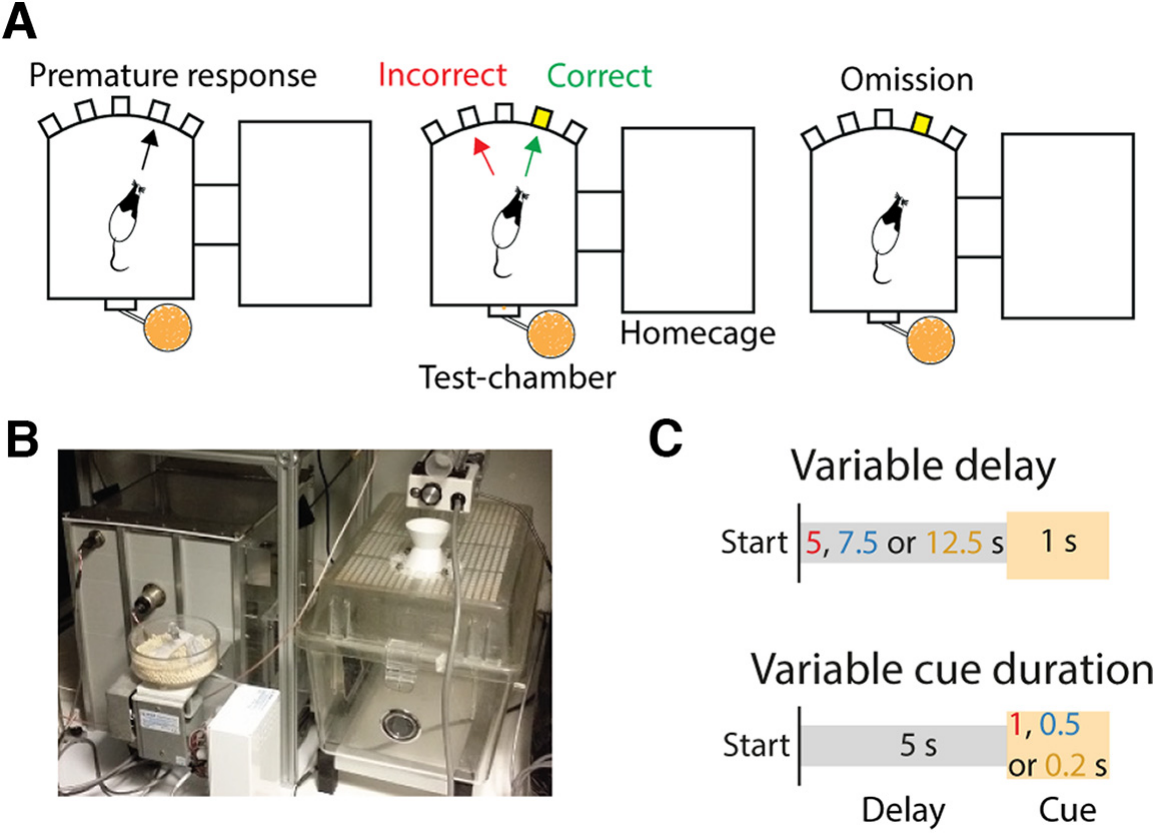
Overview of the self-paced five-choice serial reaction time task (SP-5-CSRTT) and the behavioral protocols. ***A***, Schematic of the rat CombiCage. The operant box is connected to the home-cage of the animal with a polymer tube (diameter, 10 cm). The four possible responses of a rat in a trial are shown: premature response before the onset of the cue, incorrect or correct response following the cue, or a lack of response (omission). ***B***, A photographic image depicting the rat CombiCage. ***C***, Schematic illustrations for variable delay (left) and variable cue duration (right) sessions. A complete schematic of experimental design and task stages is presented in Extended Data Figure 1-1.

Briefly, CombiCages were constructed by placing a polymer tube between a macrolon home-cage and a rat operant chamber (Fig. 1*A*,*B*; Med Associated Inc.). Animals were placed in these CombiCages 2 d before start of the training for acclimatization. Once training started, rats earned food reward pellets after correct responses in the task (Dustless Precision Pellets, grain-based, F0165, 45 mg, Bio-Serve). Additional chow was provided to maintain growth according to an 85–90% weight curve. Animals started the SP-5-CSRTT protocol with magazine training during which a pellet was delivered after a variable delay (delays: 4, 8, 16, or 32 s). In the subsequent stage the animal learned to nose poke for a reward. Now, all five cue lights were on and a nose poke in one was required for a reward. Next, the animal learned to respond to a single cue light, which was presented after a delay of 5 s. Errors were not punished at this stage.

In the final stages, the rats needed to make a nose poke in a lit cue hole after a fixed delay of 5 s. A nose poke before stimulus onset, a premature response, was punished with a 5-s time-out which was signaled by the illumination of the house light. An incorrect response, in the wrong cue hole was also punished with a time-out. Initially, the cue duration was 16 s and was titrated down to 1 s in five training stages. The limited hold period after cue presentation was 2 s. Any response after this period, or failure to respond, was considered an omission and also punished with a time-out. Correct responses resulted in the delivery of one pellet and a new trial could be started 5 s after reward collection (correct trials) or after the time-out (errors). Trials could only be started during the first 2.5 h of the dark cycle (time-restricted protocol; Bruinsma et al., 2019). To reach the subsequent training stage with shorter stimulus duration, a rat needed to start a minimum of 50 trials in the current stage, with an accuracy (ratio of correct and incorrect responses) above 80% and/or <20% omissions or performed >200 correct trials. Performance was assessed online during task performance using a sliding window of 20 trials (Remmelink et al., 2017; Bruinsma et al., 2019).

We used data from either (1) variable delay sessions, in which we randomly varied the delay between 5, 7.5, and 12.5 s within sessions or (2) variable cue duration sessions, in which we randomly varied the cue duration between 1, 0.5, and 0.2 s within sessions. These sessions were employed 3 d a week, and on days in between rats performed regular sessions with a fixed delay of 5 s and cue duration of 1 s.

### Drug administration

Clozapine N-oxide (CNO) dihydrochloride (Hello Bio) was dissolved in 0.9% saline and injected intraperitoneally 30 min before the start of the dark phase. Solutions were freshly prepared on each test day and doses were administered using a Latin square design. Animals received either 0, 5, or 10 mg/kg CNO per test day, based on previously reported CNO doses (Hart et al., 2018; Panoz-Brown et al., 2018).

### Histology and immunofluorescence

For elaborate descriptions of the histologic procedures, see de Kloet et al. (2021). Briefly, rats were anaesthetized and transcardially perfused with 4% paraformaldehyde. Coronal sections of 50 μm were sliced and stained for mCherry with rabbit anti-RFP (Rockland, 1:1000) and subsequently Alexa Fluor 546 donkey anti-rabbit (Thermo Fisher Scientific, 1:400). Images were acquired with a Nikon Eclipse Ti confocal microscope and were analyzed using ImageJ software. The area of virus expression was selected as a region of interest (ROI) in ImageJ. The area of the ROI was calculated and cells within the ROI were counted manually.

### Exclusion criteria

Animals that displayed minimal or unilateral virus expression or animals that did not learn the task to criterion performance (80% accuracy and <20% omissions) were excluded from further analysis and resulting figures/statistical analyses (de Kloet et al., 2021). This resulted in the following exclusions: insufficient unilateral viral expression: two VMS, two DMS, two MDL, four MDM rats. Did not learn task to criterion: three MDL, one MDM, one eYFP control rats (Extended Data Fig. 1-1*A*).

### Data analysis and statistics

All behavioral data were acquired with MED-PC software (Med-Associates) and analyzed using MATLAB (MathWorks). Correct, incorrect and premature responses, as well as omissions, were expressed as a percentage of total number of trials. Correct response latencies are expressed in seconds. A Shapiro–Wilk test was used to test for normal distribution of the data. For repeated measures ANOVA, the residuals were assessed for normality by inspection of the normal probability plots and χ^2^ goodness of fit tests. We excluded all trials with a magazine latency >10 s (Remmelink et al., 2017; Bruinsma et al., 2019).

**Figure 2.**
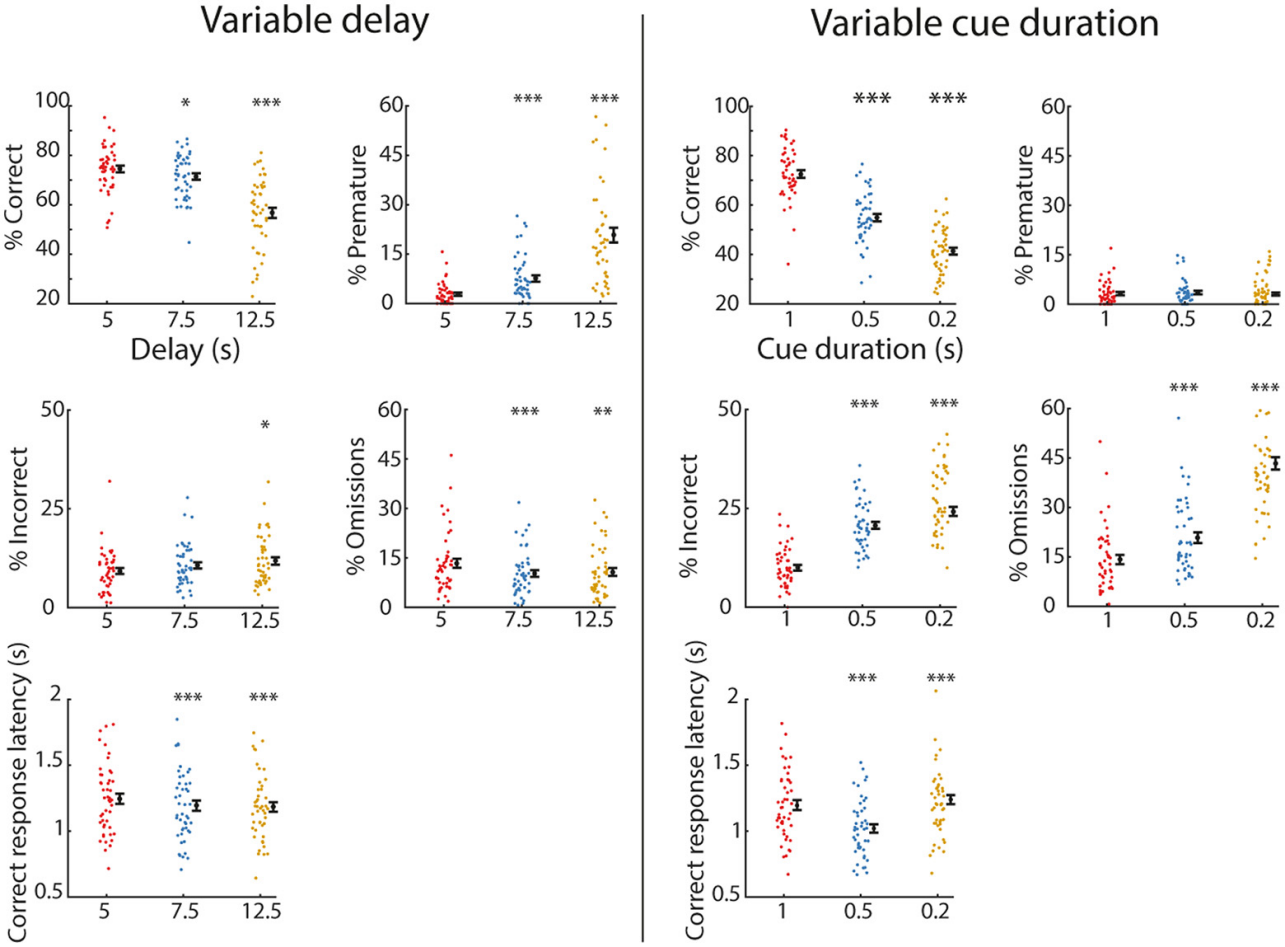
Behavioral performance and response latencies are affected by varying the trial delay and cue duration. Left, Behavioral performance in the variable delay test, with randomly varying delays of 5 s (red), 7.5 s (blue), and 12.5 s (yellow). Data are shown for percentage of correct, premature and incorrect responses, omissions, and correct response latencies. Right, Same as the left panel, but for the variable cue duration test, randomly varying cue durations of 1 s (red), 0.5 s (blue), and 0.2 s (yellow). *p < 0.05, **p < 0.01, ***p < 0.001 FDR-corrected paired *t* test or Wilcoxon signed-rank test versus delay of 5 s or cue duration of 1 s. Dots are individual rats, the black dot and error bar represent mean ± SEM. *N* = 47 animals for all sessions.

**Figure 3.**
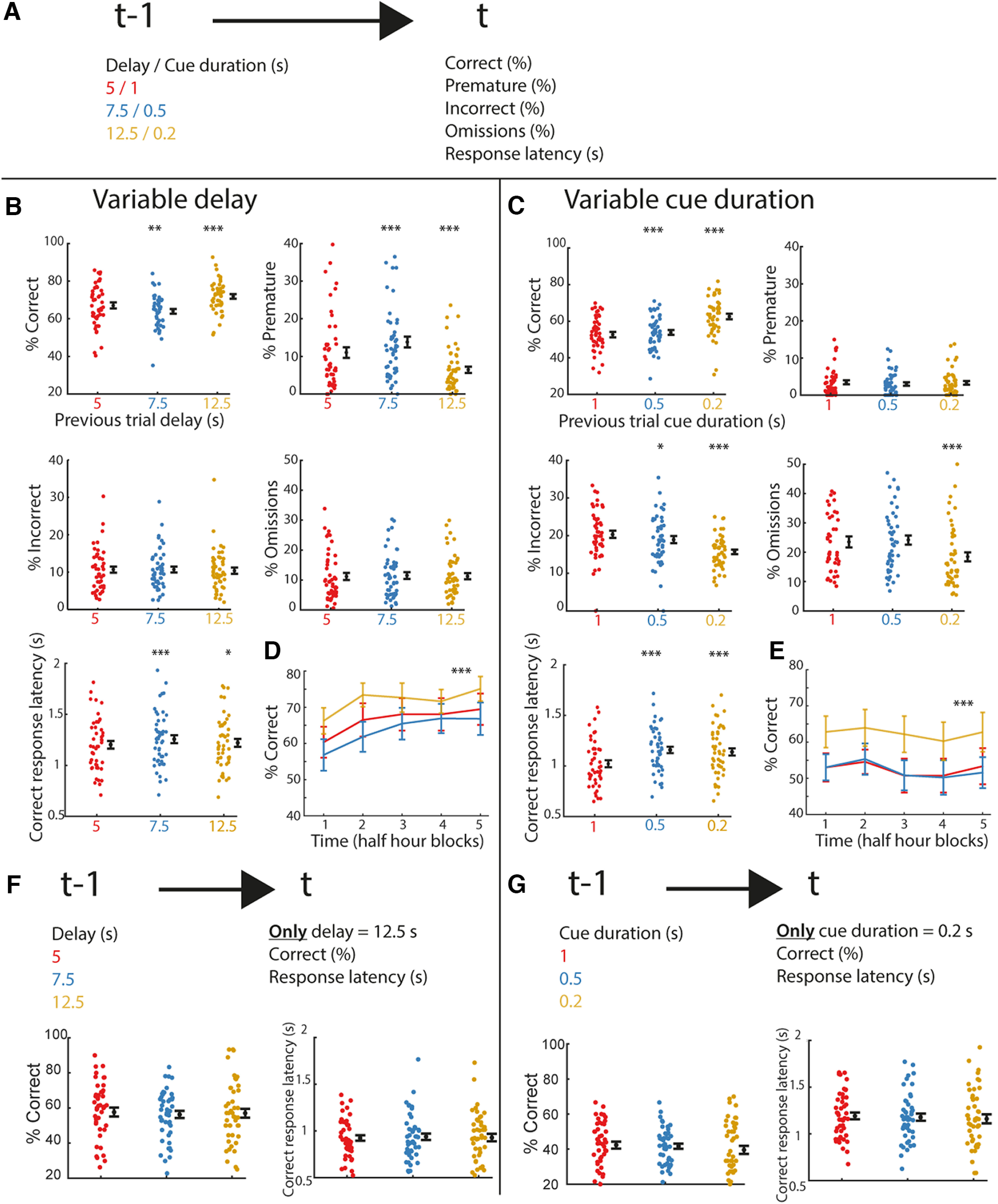
Attentional performance, inhibitory control and response latencies adapt trial-to-trial. ***A***, Schematic illustration of the analysis, effect of previous trial (t–1) delay/cue duration on current trial (t) outcome and correct response latency was analyzed. ***B***, Performance for the selected parameters in trials following those with a delay of 5 s (red), 7.5 s (blue), and 12.5 s (yellow). ***C***, Similar as ***B***, but for trials following those with a cue duration of 1 s (red), 0.5 s (blue), or 0.2 s (yellow). *p < 0.05 FDR-corrected paired *t* test versus previous delay of 5 s or cue duration 1 s. *p < 0.05, **p < 0.01, ***p < 0.001 FDR-corrected paired *t* test or Wilcoxon signed-rank versus previous delay of 5 s or cue duration 1 s. Dots are individual rats, the black dot and error bar represent mean ± SEM. ***D***, Performance after difficult trials is increased throughout entire 2.5-h session with variable delay. ***E***, Same as ***D***, for variable cue duration sessions. ***p < 0.001 main effect of delay/cue duration in repeated measures ANOVA. 95% confidence interval is plotted. ***F***, ***G***, Top, Schematic illustration of the analysis, effect of previous trial delay (***F***) or cue duration (***G***) on current trial performance and correct response latency is analyzed for only difficult trials on current trial. Bottom, Performance and correct response latency for the specific analysis. *N* = 47 animals for all sessions. All error bars are SEM except for the 95% confidence interval in panels ***D*** and ***E***.

**Figure 4.**
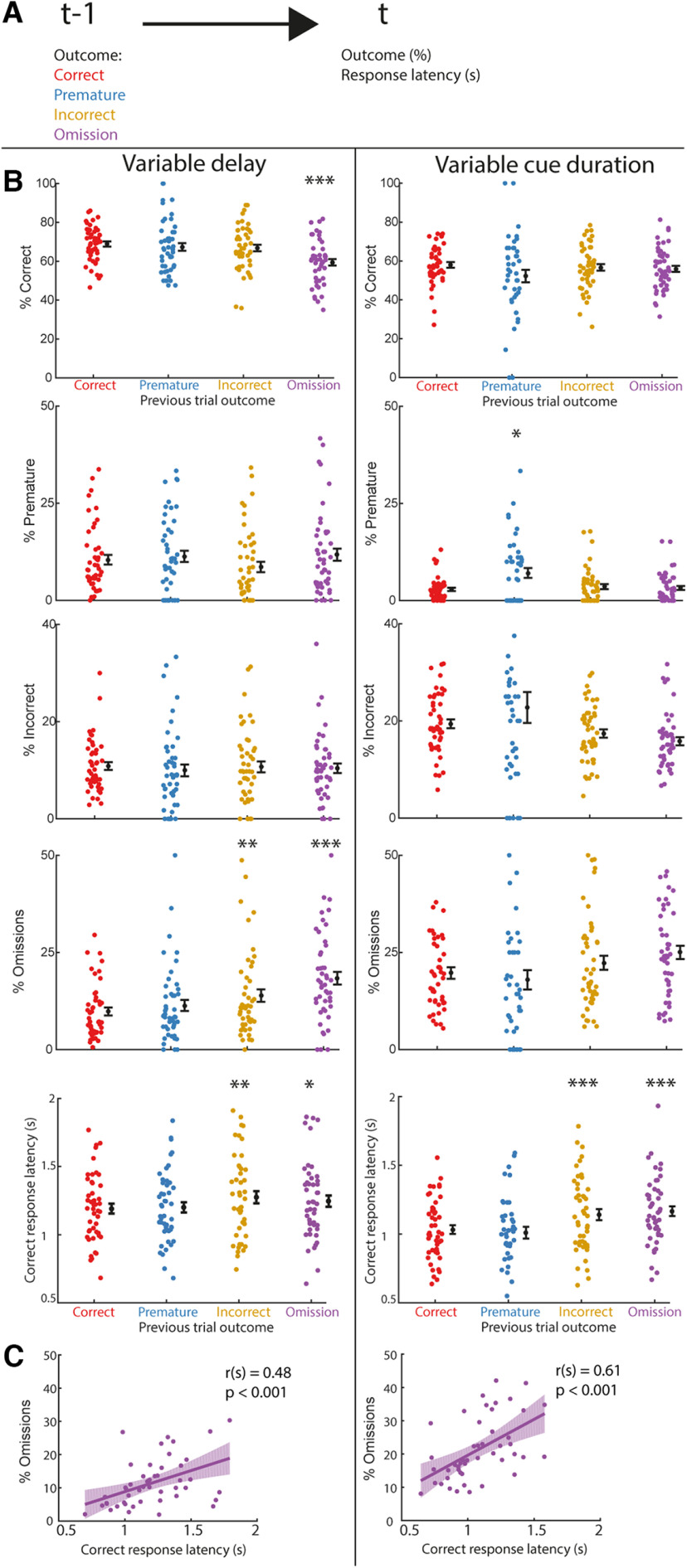
Performance decreases and responses slow down, after errors. ***A***, Schematic illustration of the analysis, effect of previous trial (t–1) outcome on current trial (t) outcome and response latency was analyzed. ***B***, Behavioral outcome parameters and correct response latency in trials after a correct trial (red), premature response (blue), incorrect trial (yellow), or omission (purple) in the previous trial. This is presented for the variable delay (left) or the variable cue duration (right) sessions. *p < 0.05, **p < 0.01, ***p < 0.001 paired *t* test or Wilcoxon signed-rank test versus correct. Dots are individual rats, the black dot and error bar represent mean ± SEM. ***C***, Correlation between correct response latency and omissions, from the variable delay (left) or variable cue duration (right) sessions. Correlation coefficients are from Spearman correlation analysis *r*(s) and are depicted together with the associated *p*-value in the graph inset. Omissions show a positive correlation with correct response latency. Dots are individual rats, line and shade represent a linear fit of the data and the associated 95% confidence interval. *N* = 47 animals for all sessions.

The effects of varying task parameters and previous trial difficulty (Figs. 2 and 3) were assessed with repeated measures ANOVAs. Mauchly’s test for sphericity was performed, lower-bound correction was applied in violation of sphericity. In case residuals were not normally distributed, a nonparametric Friedmans test was used. *Post hoc* testing was done using Wilcoxon rank-sum tests or *t* tests with Benjamin–Hochberg false discovery rate (FDR) to adjust *p* values for multiple comparisons (Benjamini and Hochberg, 1995). Effects of delay/cue duration over the 2.5-h session were analyzed by splitting the session in five blocks of 30 min. Two-way mixed repeated measures ANOVAs were used with time and delay/cue duration as within-subject factors (Fig. 3). Comparisons of behavioral parameters following a specific trial outcome (Fig. 4) were performed with repeated measures ANOVAs. Correlation coefficients (Fig. 4) were calculated using Spearman correlation analysis *r*(s).

To test the effects of CNO on adaptive control we tested for interaction effects between experimental animals expressing the DREADD receptor and control animals expressing only eYFP (Table 1; Extended Data Table 1-1; de Kloet et al., 2021). Effects on behavioral performance and latencies following an error or correct trial were assessed using two-way mixed repeated measures ANOVAs with group (MDL/MDM/eYFP or DMS/VMS/eYFP) as between-subjects factor and dose as within-subjects factor. Interactions between previous trial type (correct vs error) and CNO were tested with two-way repeated measures ANVOAs with trial type and dose as within-subjects factors. The effects of CNO on behavioral performance and latencies after previous delay or cue duration were assessed using three-way mixed repeated measures ANOVAs with dose and delay or cue duration as within-subject factors and group as between-subject factor. *Post hoc* testing was done using Wilcoxon rank-sum tests or *t* tests with Benjamin–Hochberg false discovery rate (FDR) to adjust *p* values for multiple comparisons.

**Table 1:** Only inhibition of MDL projecting mPFC neurons alters behavior after an error

Parameter	Condition	MDL	MDM	eYFP (thalamus)	DMS	VMS	eYFP (striatum)
% Correct after error variable cue duration	Saline	47.6 ± 11.81	40.49 ± 9.44	59.12 ± 11.39	52.98 ± 5.81	53.77 ± 8.52	65.91 ± 8.36
	CNO5	43.14 ± 14.4	41.1 ± 10.67	58.65 ± 10.28	51.55 ± 5.38	52.34 ± 11.66	63.66 ± 7.03
	CNO10	38.54 ± 12.71	38.2 ± 13.33	60.59 ± 12.18	51.87 ± 4.23	49.46 ± 9.30	62.50 ± 6.60
Correct response latency (s) after error variable cue duration	Saline	0.99 ± 0.22	1.08 ± 0.32	0.74 ± 0.19	1.24 ± 0.15	1.21 ± 0.31	1.07 ± 0.16
	CNO5	1.03 ± 0.23	0.99 ± 0.25	0.75 ± 0.19	1.20 ± 0.15	1.17 ± 0.30	1.06 ± 0.17
	CNO10	1.04 ± 0.24	1.01 ± 0.30	0.72 ± 0.18	1.20 ± 0.14	1.14 ± 0.32	1.08 ± 0.12
% Correct after error variable delay	Saline	56.56 ± 10.98	55.84 ± 8.56	66.11 ± 12.88	69.08 ± 4.41	59.66 ± 7.05	67.84 ± 9.65
	CNO5	58.69 ± 13.79	48.81 ± 9.29	68.06 ± 11.92	66.55 ± 5.14	58.81 ± 8.84	66.78 ± 9.89
	CNO10	50.94 ± 13.57	49.74 ± 11.38	68.72 ± 11.58	65.55 ± 7.18	60.52 ± 7.84	65.62 ± 6.73
Correct response latency (s) after error variable delay	Saline	1.02 ± 0.18	1.19 ± 0.30	0.82 ± 0.22	1.24 ± 0.15	1.31 ± 0.37	1.12 ± 0.18
	CNO5	1.04 ± 0.18	1.10 ± 0.23	0.83 ± 0.22	1.19 ± 0.16	1.25 ± 0.28	1.11 ± 0.14
	CNO10	1.14 ± 0.19**	1.14 ± 0.25	0.83 ± 0.21	1.17 ± 0.12	1.22 ± 0.29	1.09 ± 0.16

Percentage of correct responses and the correct response latency in seconds is presented in trials following error trials. Sessions in which animals received saline injections are compared with sessions where they received CNO 5 mg/kg (CNO5) or CNO 10 mg/kg (CNO10). Data are presented as mean ± SD. Interactions between thalamus (MDL/MDM/eYFP) and striatum (DMS/VMS/eYFP) groups are tested using a two-way mixed repeated measures ANOVAs with group (MDL/MDM/eYFP or DMS/VMS/eYFP) as between-subjects factor and dose as within-subjects factor. **p < 0.01 FDR-corrected paired *t* test versus saline. MDL *n* = 11, MDM *n* = 11, eYFP (thalamus) *n* = 13, DMS *n* = 10, VMS *n* = 12, eYFP (striatum) *n* = 13 rats. Additional data of performance after specific delay or cue duration trials are presented in Extended Data Table 1-1.

In all cases, the significance level was set at *p *<* *0.05. Data are presented as mean ± SEM throughout the main text and figures, unless stated otherwise.

## Results

### Variable delay and cue duration affect behavioral performance

Long periods of training can lead to overtraining of animals in operant tasks, resulting in a shift from cognitive, goal-directed behavior to habitual behavior (Jog et al., 1999; Yin and Knowlton, 2006; Smith and Graybiel, 2016). Since rats performed hundreds of trials during daily 2.5-h sessions in the self-paced version of the five-choice serial reaction time task (SP-5-CSRTT; Fig. 1), standard sessions with fixed delay and cue duration favor habitual behavior. To prevent rats from relying on habitual behavior during the task, cognitively challenging sessions were used on testing days (Bruinsma et al., 2019). For this, as described above in Materials and Methods, we have used sessions during which we randomly varied the delay before the onset of the cue, and sessions during which the cue duration was randomly varied (Fig. 1*C*). Based on literature, we expected the former manipulation to increase premature responding, whereas the latter manipulation should affect attentional parameters (Bari et al., 2008; Remmelink et al., 2017; Bruinsma et al., 2019). Here, we present re-analyzed data from previously published experiments (de Kloet et al., 2021).

During variable delay sessions (delays: 5, 7.5, and 12.5 s), animals (*n* = 47) completed 392 trials on average, with a standard deviation of 75 trials. Longer delays increased the percentage of premature responses (*F*_(2,92)_ = 81.94, *p* < 0.001; Fig. 2, left) and reduced the percentage of correct responses (*F*_(2,92)_ = 52.44, *p* < 0.001). We also observed a small increase in incorrect responses in trials with a delay of 12.5 s (*F*_(2,92)_ = 6.51, *p* = 0.002). Finally, small decrements were observed in the percentage of omissions (χ^2^(2) = 22.21, *p* < 0.001) and in correct response latencies (*F*_(2,92)_ = 13.76, *p* < 0.001).

During variable cue duration sessions (durations: 1, 0.5, and 0.2 s) animals completed on average 449 trials, with a standard deviation of 99 trials. Shortening the cue duration decreased the percentage of correct responses (*F*_(2,92)_ = 416.11, *p* < 0.001; Fig. 2, right) accompanied by both increased incorrect responses (*F*_(2,92)_ = 133.83, *p* < 0.001) and omissions (*F*_(2,92)_ = 193.02, *p* < 0.001). Manipulating the cue duration did not affect premature responding (*F*_(2,92)_ = 1.43, *p* = 0.24). Finally, we observed a decrease in correct response latency when cue durations were shortened (*F*_(2,92)_ = 96.78, *p* < 0.001).

### Trial difficulty history affects inhibitory control, attentional performance, and response latency

In both rodents and human subjects, the history of reward, choice and sensory stimulus information has been shown to impact current behavioral output (Abrahamyan et al., 2016; Hwang et al., 2017; Akrami et al., 2018). We tested whether the history of trial difficulty affects performance in the SP-5-CSRTT. We hypothesized that animals adapt behavior following difficult trials, that is trials with a lengthened delay or a shortened cue duration, in a similar fashion to behavioral updates after error trials (de Haan et al., 2018).

First, we tested the effect of previous trial delay on current trial performance in the variable delay protocol (Fig. 3*A*). Animals showed a small decrease in correct responding after a delay of 7.5 s and an increase in correct responses after the longest delay of 12.5 s (*F*_(2,92)_ = 27.00, *p* < 0.001; Fig. 3*B*). Concomitant to the increase in correct responses after the longest delay, animals showed a decrease in premature responses (χ^2^(2) = 60.10, *p* < 0.001). In contrast, previous trial delay had no effect on incorrect responses (*F*_(2,92)_ = 0.14, *p* = 0.87) or omissions (*F*_(2,92)_ = 0.14, *p* = 0.87). Finally, response latencies were longer after trials with a long delay (*F*_(2,92)_ = 20.31, *p* < 0.001).

Next, we tested the effect of previous trial cue duration on current trial performance in the variable cue duration protocol. There was an increase in correct responses in trials following a short cue duration (*F*_(2,92)_ = 53.8, *p* < 0.001; Fig. 3*C*). This was associated with both a decrease in incorrect responses (*F*_(2,92)_ = 26.08, *p* < 0.001) and omissions (*F*_(2,92)_ = 17.2, *p* < 0.001). Premature responding was not affected by previous trial cue duration (*F*_(2,92)_ = 1.08, *p* = 0.34). Lastly, previous trial cue duration increased correct response latencies (*F*_(2,92)_ = 76.32, *p* < 0.001).

We questioned whether this behavioral adaptation of correct performance is present throughout the entire 2.5-h session or whether gradual learning effects occur. To this aim, we divided the sessions in five bins of 30 min and tested for interactions between the effects of previous trial delay/cue duration and time bins on performance. Animals adapted their behavior after difficult trials throughout the entire session since no interaction effect with time was observed. This was the case for both previous trial delay (delay: *F*_(2,92)_ = 28.19, *p* < 0.001, time: *F*_(4,184)_ = 9.41, *p* < 0.001, delay × time: *F*_(8,368)_ = 0.87, *p* = 0.54; Fig. 3*D*), as well as previous trial cue duration (cue duration: *F*_(2,92)_ = 52.81, *p* < 0.001, time: *F*_(4,184)_ = 2.32, *p* = 0.058, cue duration × time: *F*_(8,368)_ = 0.25, *p* = 0.98; Fig. 3*E*).

One possibility is that animals only perform better, and slow their responses, when a trial is preceded by a more difficult one. To test this, we performed the same analysis, but now only selected difficult trials with a delay of 12.5 s or a cue duration of 0.2 s for the current trial. On these difficult current trials, previous trial delay had no effect on both the performance (*F*_(2,92)_ = 0.28, *p* = 0.60; Fig. 3*F*) as well as the correct response latencies (*F*_(2,92)_ = 0.79, *p* = 0.38). Also in the variable cue duration protocol, current difficult trials, with a cue duration of 0.2 s, both performance (*F*_(2,92)_ = 1.74, *p* = 0.19; Fig. 3*G*) and the response latencies (*F*_(2,92)_ = 0.1, *p* = 0.76) were not affected by previous trial difficulty.

Together, these data suggest that animals adapt behavior after cognitively demanding trials to optimize behavioral performance in subsequent easier trials, however, performance was similar when followed by a difficult trial.

### Posterror slowing of responses

More difficult trials lead to more error responses (Fig. 2) and it has previously been shown that animals adapt behavior after an error response (Pinto and Dan, 2015; De Haan et al., 2018; Norman et al., 2021). How trial-to-trial performance after specific trial outcomes is regulated in the 5-CSRTT is not known. Therefore, we analyzed the effect of previous trial outcome on current trial outcome and the correct response latency (Fig. 4*A*).

In the protocol with variable delays, we observed a decrease in correct responses after omissions only (*F*_(2,92)_ = 11.4, *p* < 0.001; Fig. 4*B*, left). We saw an increase in omitted trials following incorrect responses or omissions (χ^2^(3) = 13.30, *p* < 0.001). However, premature (*F*_(2,92)_ = 3.15, *p* = 0.08) or incorrect responses (*F*_(2,92)_ = 0.22, *p* = 0.64) were not modulated by previous trial outcome. Additionally, correct response latencies increased after an incorrect responses and omissions, indicating slower responses (*F*_(2,92)_ = 6.55, *p* < 0.001).

In the protocol with variable cue durations, we saw no decrease in correct responses following an error (*F*_(2,92)_ = 1.72, *p* = 0.2; Fig. 4*B*, right). However, we found a small increase in premature responses, when preceded by a premature response (*F*_(2,92)_ = 7.45, *p* < 0.01). Incorrect responses (*F*_(2,92)_ = 3.28, *p* = 0.08) and omissions (*F*_(2,92)_ = 3.54, *p* = 0.07) were not altered by previous trial outcome. Similar as in the variable delay protocol, correct response latencies were increased after incorrect responses and omissions (*F*_(2,92)_ = 12.60, *p* = 0.001).

In both protocols, animals slowed their responses and made more omissions in the variable delay protocol after attentional errors, incorrect responses and omissions. We performed correlation analyses to look at the relationship between these two parameters in more detail. We found that there was a positive correlation between correct response latency and omissions in both the variable delay [*r*(s) = 0.48, *p* < 0.001; Fig. 4*C*, left] and the variable cue duration session [*r*(s) = 0.61, *p* < 0.001; Fig. 4*C*, right]. This suggests that slower animals omit more trials in the task.

In summary, we show that animals slow their responses after attentional errors and make more omissions following omitted trials in the variable delay protocol.

### MDL projecting neurons in the mPFC are involved in posterror adaptive control

What is the neuronal circuitry mediating adaptive control of behavior? Prefrontal regions mediate trial-by-trial behavioral adaptation in both human subjects and rats (Sheth et al., 2012; Narayanan et al., 2013). Recently, optogenetic stimulation of prefrontal projections to the visual cortex increased performance after errors in the 5-CSRTT (Norman et al., 2021). However, it is not known whether prefrontal projections to the thalamus or to the striatum play a role in regulating this behavior.

**Figure 5.**
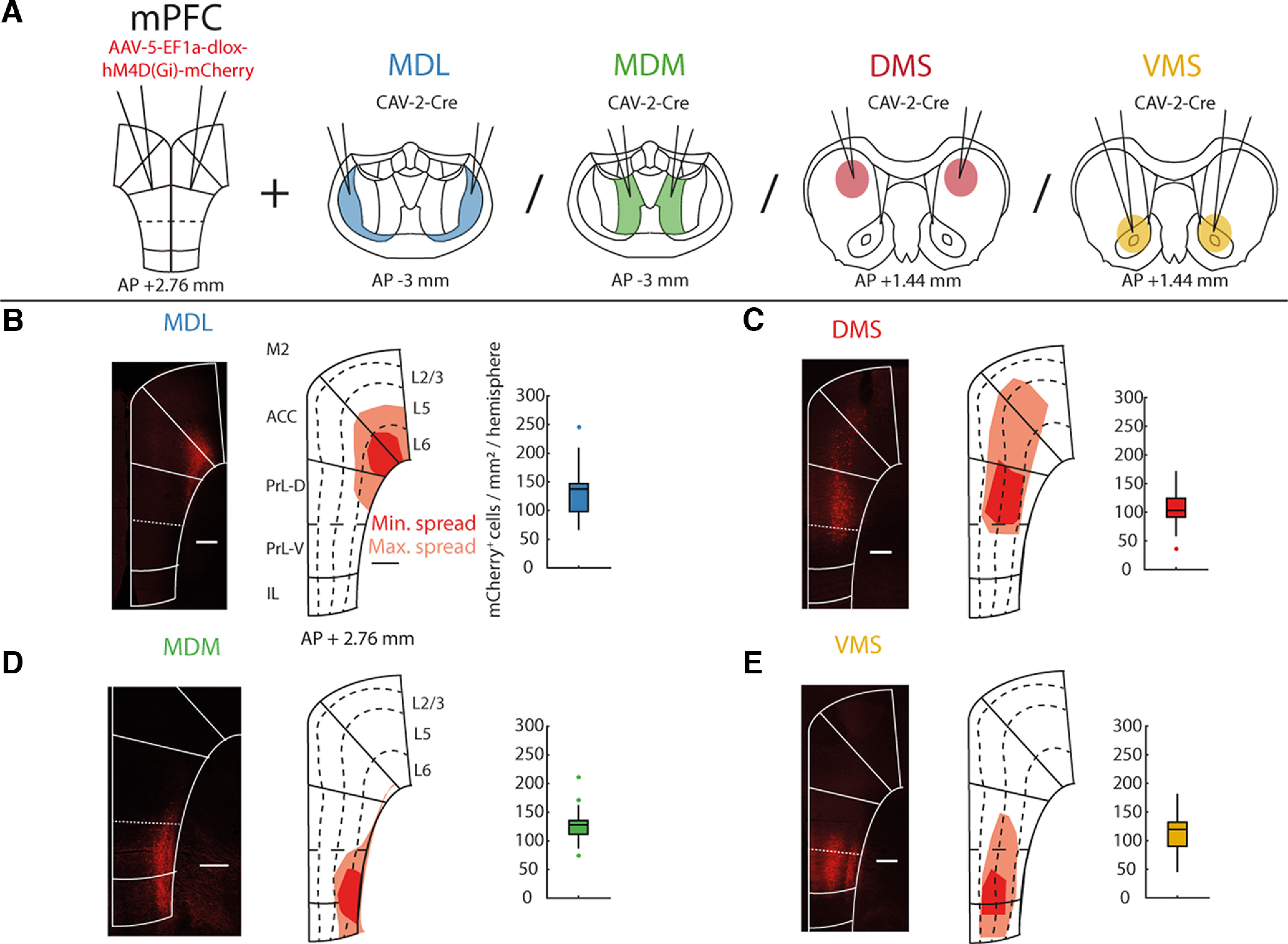
Expression of the inhibitory hM4D(Gi) DREADD receptor in specific prefrontal projection populations. ***A***, Schematic experimental design for the expression of the inhibitory hM4D receptor specifically in mPFC projection neurons. This experiment is already published, see de Kloet et al. (2021) for details. ***B***, Histologic example (left), schematic representation of the spread of virus expression (middle) and quantified number of mCherry positive cells (right) for animals expressing hM4D specifically in neurons in dorsal mPFC projecting to the MDL. ***C–E***, Similar as ***B***, but for animals expressing hM4D in neurons in mPFC projecting to DMS, MDM, and VMS, respectively. Scale bar is 500 μm. MDL *n* = 11 (22 hemispheres), DMS *n* = 10 (20 hemispheres), MDM *n* = 11 (22 hemispheres), VMS *n* = 12 (24 hemispheres). Boxplots represent median and the 25th and 75th percentiles.

To test this, we expressed inhibitory hM4D(Gi) DREADD (Designer Receptor Exclusively Activated by Designer Drugs) receptors in projection neurons of the mPFC that project specifically to either the medial or lateral nucleus of the MDT (MDM and MDL, respectively) or to the dorsal or ventral striatum (DMS and VMS, respectively). For this, we infused the retrograde virus CAV-2-Cre in one of the four subcortical target areas and the AAV virus with a floxed hM4D(Gi) cassette in the mPFC (Fig. 5*A*). We observed robust expression of the DREADD receptor in the mPFC for all projection areas (Fig. 5B–E). The same animals were used to test inhibition of mPFC projection neurons on SP-5-SCRTT behavior in both the variable delay and cue duration sessions (de Kloet et al., 2021), and we here analyzed the behavioral data to test the role of mPFC projection neurons in behavioral adaptation. We also confirmed the presence of axonal fibers of mPFC projection neurons in the target areas and confirmed anatomic injection locations with retrograde tracer experiments (de Kloet et al., 2021). Here, we pooled the error trials to keep statistical power, since our group size for these chemogenetic experiments was smaller (*N* = 10–13 rats).

**Figure 6.**
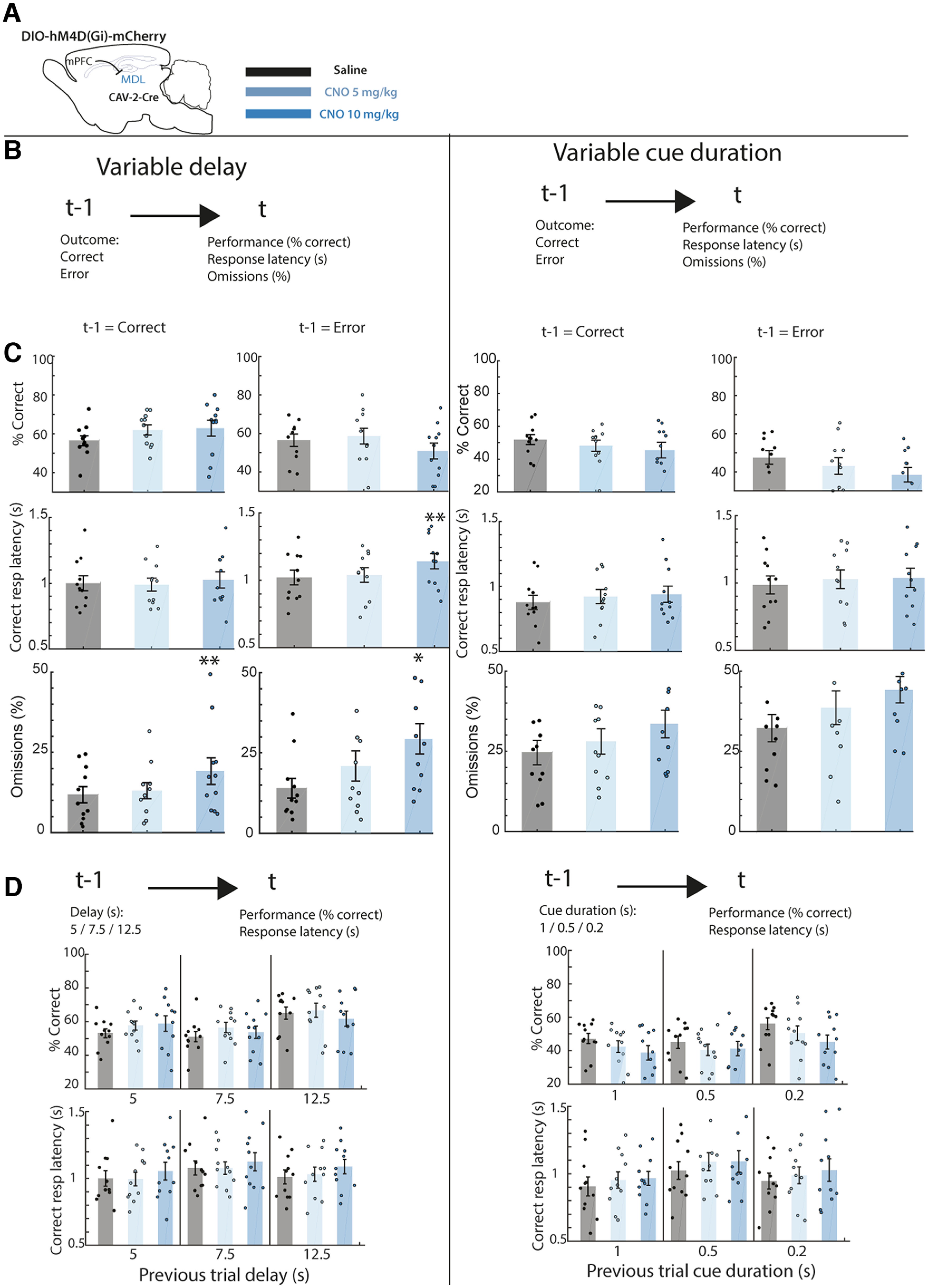
Chemogenetic inactivation of MDL-projecting mPFC neurons alters posterror adaptive control of responses. ***A***, Schematic experimental design for the expression of the hM4D(Gi) DREADD receptor specifically in MDL-projecting neurons (left) and the legend for the CNO doses used (right). See de Kloet et al. (2021) for more details and results from the experiment. ***B***, Schematic illustration of the analysis, the performance (% correct), correct response latency or the omissions were analyzed after error or correct trials and compared for sessions in which saline or CNO was administered. ***C***, Variable delay sessions (left): CNO increases posterror response latencies specifically after error trials and causes a larger increase in omissions compared with after correct trials. Variable cue duration sessions (right): CNO does not specifically alter performance after error trials**. *D***, Top, schematic illustration of the analysis, the performance or response latency was analyzed after a trial with a random delay (left) or cue duration (right) and compared for sessions in which either saline or CNO was administered. CNO did not alter behavioral response after a delay or cue duration. **p < 0.01 FDR-corrected paired *t* test versus saline. Dots are individual rats, bar graphs represent mean ± SEM. *N* = 11 rats.

In the variable delay protocol, when we inhibited corticothalamic projection neurons we found an interaction effect on the percentage of correct responses after an error (Fig. 6*C*, left**;** Table 1, group × dose: *F*_(4,64)_ = 3.83, *p* = 0.007). However, subsequent *post hoc* testing did not reveal specific deficits in posterror behavioral performance. Inhibiting mPFC to MDL projections did increase posterror slowing of responses (Fig. 6*C*, left; Table 1, group × dose: *F*_(4,64)_ = 6.84, *p* < 0.001). This effect was specific for posterror trials and not observed after correct responses (Fig. 6*C*, left, previous trial × dose: *F*_(4,10)_ = 8.67, *p* < 0.01). Inhibiting mPFC to MDM projections did not affect posterror slowing, nor did CNO injection in eYFP control animals (Table 1).

Since CNO increased posterror slowing in the mPFC to MDL group, we wondered how omissions were affected after an error, since correct response latencies and omissions are correlated (Fig. 4*C*) and omissions increase after mPFC to MDL inhibition (de Kloet et al., 2021). Here, mPFC to MDL inhibition increased omissions after correct trials, but this effect was more pronounced after error trials (dose: *F*_(4,10)_ = 12.66, *p* < 0.01; previous trial × dose: *F*_(4,10)_ = 3.99, *p* < 0.05; Fig. 6*C*, left). Finally, this effect of CNO on the omissions was specific to the MDL group and omissions were not altered by CNO in either the eYFP or MDM group (group × dose: *F*_(4,64)_ = 4.68, *p* < 0.01).

We next tested the role of corticothalamic projection neurons in behavioral adaptation in trials following trials with short or long delay. We found no effect of administering CNO on both percentage of correct trials after a long delay in the previous trial (Fig. 6*D*, left; Extended Data Table 1-1, group × dose × delay: *F*_(8,128)_ = 1.48, *p* = 0.17) or on correct response latency after a long delay (group × dose × delay: *F*_(8,128)_ = 0.58, *p* = 0.79).

Subsequently, we inhibited the corticothalamic projection neurons in the variable cue duration sessions and analyzed the effect of CNO on behavioral adaptation. Inhibition of MDL-projecting neurons decreased the percentage of correct responses after an error (Fig. 6*C*, right; Table 1 group × dose: *F*_(4,64)_ = 2.97, *p* = 0.026). However, this effect was not specific for posterror responses (dose: *F*_(4,10)_ = 7.28, *p* < 0.05; previous trial × dose: *F*_(4,10)_ = 0.27, *p* = 0.77; Fig. 6*C*, right). Inhibition of MDM-projecting neurons or eYFP control animals (Table 1) did not lead to alterations in posterror performance. We found an interaction effect on the correct response latency after an error (Fig. 6*C*, right; Table 1, group × dose: *F*_(4,64)_ = 3.71, *p* = 0.009). However, subsequent *post hoc* testing did not reveal any effects of CNO on posterror latencies in the different groups. Analysis of the percentage of omitted responses revealed a specific increase of omissions in the mPFC to MDL group (group × dose: *F*_(4,64)_ = 4.72, *p* < 0.01; Fig. 6*C*, right). However, this effect was not specific to trial type, suggesting that the animals made more omissions after both correct and error trials (dose: *F*_(4,10)_ = 16.13, *p* < 0.01; previous trial × dose: *F*_(4,10)_ = 0.71, *p* = 0.50; Fig. 6*C*, right).

Finally, we analyzed whether inhibition of corticothalamic neurons altered behavioral adaptation following trials with a long or short cue duration. We found no effect of administering CNO on both percentage of correct trials after a certain cue duration in the previous trial (Fig. 6*D*, right; Extended Data Table 1-1, group × dose × cue duration: *F*_(8,128)_ = 0.87, *p* = 054) or on correct response latency after a certain cue duration (group × dose × cue duration: *F*_(8,128)_ = 0.95, *p* = 0.80).

In summary, when we inhibited MDL-projecting neurons in the dorsal mPFC we found that animals made slower responses after an error in the variable delay sessions and made more omissions, and effect that was more pronounced after error trials.

### Corticostriatal projection neurons in the mPFC are not involved in adaptive control

We next tested whether corticostriatal projections neurons, projecting to the anterior DMS or VMS, were involved in the adaptive control of behavior following an error or difficult trials.

In the variable delay sessions, inhibiting corticostriatal projection neurons with CNO had no effect on either the percentage of correct responses (Table 1, group × dose: *F*_(4,62)_ = 0.77, *p* = 0.55) or the correct response latency following an error (Table 1, group × dose: *F*_(4,62)_ = 0.48, *p* = 0.75). Additionally, we found no effect of inhibiting cortico-striatal populations on the percentage of correct response after long delays (Extended Data Table 1-1, group × dose × delay: *F*_(8,124)_ = 0.85, *p* = 0.55) or on the correct response latency following long delays (Extended Data Table 1-1, group × dose × delay: *F*_(8,124)_ = 0.44, *p* = 0.90).

Subsequently, we analyzed the effect of CNO on behavioral adaptation in the variable cue duration sessions. Inhibiting corticostriatal projections had no effect on the performance (Table 1, group × dose: *F*_(4,62)_ = 0.41, *p* = 0.80) or correct response latency (Table 1, group × dose: *F*_(4,62)_ = 0.76, *p* = 0.55) following an error. Finally, we found no effect of CNO on the performance (Extended Data Table 1-1, group × dose × cue duration: *F*_(8,124)_ = 0.85, *p* = 0.56) or the correct response latency (Extended Data Table 1-1, group × dose × cue duration: *F*_(8,124)_ = 1.01, *p* = 0.43) after difficult trials with shorter cue durations.

Taken together, we found that while inhibiting mPFC projection neurons to medial MDT and to striatum did not affect adaptive behavior, the mPFC to MDL projection neurons are involved in the trial-by-trial adaptation of behavior by posterror slowing of responses and increasing omissions, especially after error trials.

## Discussion

In this study, we found that rats adapt their behavior on a trial-by-trial basis and we uncovered neuronal circuits in the mPFC underlying this phenomenon. We find that rats slow their responses after errors and after difficult trials. Specifically, when delay durations were unpredictable, performance is decreased after attentional errors, increasing omissions. Using a combination of retrograde labeling and chemogenetics, our findings reveal that mPFC neurons projecting to the lateral portion of the mediodorsal thalamus (MDL) regulate the adaptive control of posterror response speed.

To our knowledge, this is the first time that rats have been shown to adapt response latencies and behavioral performance following cognitively demanding trials in the 5-CSRTT. As such, this seems reminiscent of the Gratton effect found in human studies, that is human participants have been found to slow responses after incongruent or difficult trials, and depending on the exact task, increase behavioral performance (Gratton et al., 1992; Sheth et al., 2012; Bartoli et al., 2018). Interestingly, rats adapted their behavior and response speed when we increased the cognitive load in the task taxing either attentional processing or inhibitory control, suggesting that the modality of the cognitive load is not the determining factor in this adaptive control. Interestingly, this effect was only present when the difficult trial was followed by an easier trial.

As observed in previous human and rodent studies, we found a posterror slowing of responses in sessions during which we varied either the delay or cue duration (Rabbitt, 1966; Narayanan et al., 2013). This posterror slowing was not accompanied by an increase in correct responses; in contrast, animals performed worse, in line with previous data from the 5-CSRTT (Norman et al., 2021). Small improvements in behavioral performance after an error have been observed in rodent operant tasks (Pinto and Dan, 2015; De Haan et al., 2018). Whereas intuitively a posterror slowing of responses would seem to improve accuracy, human studies have shown that these parameters are not always correlated (Danielmeier and Ullsperger, 2011; Ullsperger et al., 2014). Indeed, work in nonhuman primates and human subjects showed that posterror slowing was explained by both an increased response threshold and a decreased sensitivity to sensory information (Purcell and Kiani, 2016; Ullsperger and Danielmeier, 2016). Possibly, the decreased sensitivity to sensory information is reflected in the increase in omissions we observe following errors.

**Figure 7.**
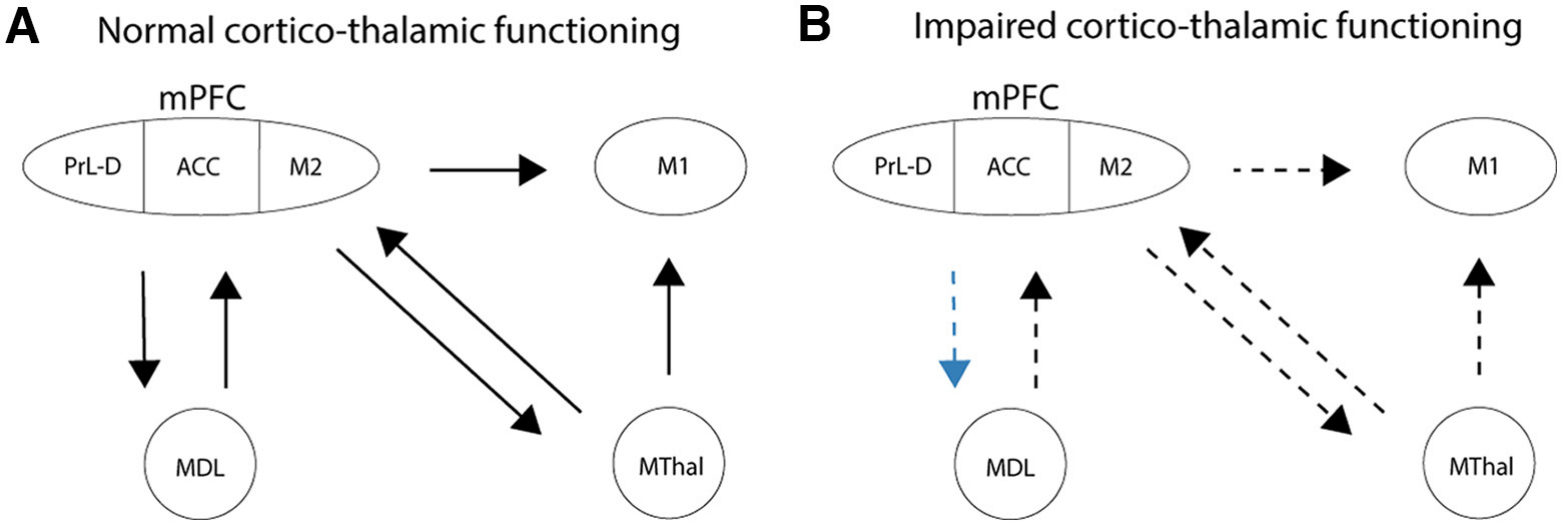
Hypothesized network underlying the behavioral changes after mPFC-MDL inhibition. ***A***, In normal situations, reciprocal mPFC – MDL connectivity maintains a rule representation for responding on a trial in the 5-CSRTT. Responses are guided via projections of dorsal mPFC directly to MThal and M1. ***B***, Inhibition of MDL projecting neurons in the mPFC decreases mPFC output to MThal and likely alters local connectivity in dorsal mPFC via reciprocal connections from MDL. Reduced or disturbed output from dorsal mPFC to MThal and M1 can increase response latencies and omissions and decrease premature responses (de Kloet et al., 2021).

As for the neuronal substrates of posterror performance, a recent study showed that 30-Hz optogenetic stimulation of ACC neurons projecting to the visual cortex increases posterror performance in the 5-CSRTT (Norman et al., 2021). We only observed an effect on both posterror slowing and omissions when we inhibited a mPFC population that projects to the MDL. Recently, it has been shown that MDT projecting neurons in the mPFC maintain representations of outcome feedback (Spellman et al., 2021). Previously, we have shown that MDL projecting neurons are located in the deep layers of dorsal mPFC, mainly in the secondary motor cortex (M2) and in the ACC (de Kloet et al., 2021). Single neuron recordings show posterror increases in firing rates especially in dorsal mPFC and ACC that persist in the following trial (Narayanan and Laubach, 2008; Totah et al., 2009; Pinto and Dan, 2015). Pharmacological inhibition of dorsal mPFC abolishes posterror slowing (Narayanan and Laubach, 2008). Additionally, it was shown that inhibition of dorsal mPFC reduced delay-activity of the primary motor cortex (M1) and led to a loss of the increased phase locking of spikes and local field potentials in this region in posterror trials (Narayanan and Laubach, 2006; Narayanan et al., 2013). M1 inhibition has been proposed as a neural mechanism for posterror slowing and abnormal connectivity between the PFC and M1 has recently been shown to underlie motor inhibition deficits in schizophrenia (Ullsperger et al., 2014; Du et al., 2019). Based on our data, and the abovementioned studies, we hypothesize that effects on posterror slowing after dorsal mPFC manipulation might be actualized via modulation of M1 activity (Fig. 7).

How does the mPFC modulate M1 activity and how are MDL projecting neurons involved? Dorsal mPFC, especially M2, projects to M1 (Sesack et al., 1989; Vertes, 2006). Additionally, the MDL sends projections to M1, although this has been shown to be mainly to vibrissal motor cortex, unlikely to be involved in adaptive control (Miyashita et al., 1994; Wang and Shyu, 2004). Three other possibilities arise. First, MDL has been shown to reciprocate mPFC input (Collins et al., 2018). Since the MDL has been shown to sustain or modulate task-related mPFC activity, mPFC to MDL projections might reciprocally sustain or modulate ACC/M2 activity, and thereby control M1 activity (Schmitt et al., 2017; Rikhye et al., 2018). Second, dorsal mPFC and MDL could directly or indirectly modulate subthalamic nucleus activity, which is involved in motor control and impulsivity (Heidbreder and Groenewegen, 2003; Dalley and Robbins, 2017). Finally, MDL projecting neurons in the dorsal mPFC have been shown to send collaterals to motor thalamus (Collins et al., 2018). Inhibition of MDL projecting neurons in the mPFC might therefore disrupt motor thalamus output to M1 or reciprocal activity of motor thalamus back to mPFC (Fig. 7). In the current experiments, we only manipulated mPFC projection neurons. A more detailed study of this network during behavior is necessary to shine more light on these possible hypotheses. Additionally, whether the network involving dorsal mPFC to MDL and ACC to visual cortex involve similar mechanisms of posterror modulation of behavior or whether these circuits operate independently is still an open question (Norman et al., 2021). Why effects of this manipulation were only observed in the variable delay condition remains an interesting question for future research. We observed effects of mPFC–MDL inhibition on premature responses also exclusively in the variable delay sessions (de Kloet et al., 2021). It has been suggested that mPFC–MDT interactions maintain rule representations (Schmitt et al., 2017). Possibly, the interaction between these areas is more involved in rules that apply to the variable delay session compared with the variable cue duration session.

The dorsal mPFC also projects to the DMS. Whereas we found a role of DMS projecting neurons in cognitive control, here we do not find any effect of inhibiting these neurons on adaptive behavior (Terra et al., 2020; de Kloet et al., 2021). A recent study demonstrated that inhibition of DMS projecting neurons in the mPFC led to impairments in action selection based on recent reward history (Bari et al., 2019). Crucially, animals in this task needed to make a decision based on reward history of recent choices. Neuronal circuitry may differ from tasks that require a decision-making component versus stimulus response-based tasks. For instance, it has been shown that impulsive decision-making and impulsive action are distinct phenomena in rats and humans with partly different underlying neuronal mechanisms (Broos et al., 2012; Dalley and Robbins, 2017).

We found no effect of inhibiting VMS projecting neurons in adaptive control. Here, we inhibited projections from the ventral mPFC to the medial nucleus accumbens core and the ventral caudate putamen (de Kloet et al., 2021). There is another projection from the dorsal mPFC, which is more connected to the lateral part of the nucleus accumbens core (Heidbreder and Groenewegen, 2003; Voorn et al., 2004). Since mainly the dorsal mPFC is involved in adaptive control, it could follow that its ventral striatal output, the lateral nucleus accumbens core is additionally involved in this type of control instead of the medial region, which is more connected to ventral mPFC. More studies are needed to elucidate the role of mPFC-ventral striatal pathways in adaptive control of behavior.

Whereas we found a role for corticothalamic projection neurons in posterror adaptive control, we did not find any effect of mPFC projection neuron manipulations on adaptation of performance or response speed after difficult trials. Recent studies have implicated the posterior parietal cortex (PPC) as a critical region for processing history of sensory stimuli and using that to bias action selection (Hwang et al., 2017; Akrami et al., 2018). M2 is connected with the PPC and these projections would be an interesting candidate for the adaptive control after difficult trials (Reep et al., 1994).

Finally, there are some limitations to the current study that need to be highlighted. We did not perform a staining of cre-recombinase to confirm placement in target area. Additionally, data supported by large amounts of *in vivo* recorded projection neurons, or trial-by-trial control of neuronal activity by optogenetics would lead to more insight in the role of these projection populations in adaptive control of behavior. Finally, video recordings of rats performing the task would have led to more information about when the animals paid attention to the cues. Now, the question remains if omissions were because of attentional errors or because of nontask behaviors of the animals.

To our knowledge, this is the first time that adaptive control of behavior, and the specific contributions of these prefrontal projection populations, has been analyzed in a trial-by-trial fashion. In conclusion, we show that rats adapt their behavior on a trial-by-trial basis and that a specific projection population in the mPFC that projects to the MDL is involved in this adaptive control.

10.1523/ENEURO.0254-22.2022.t1-1 Extended Data Table 1-1 Inhibition of mPFC projection neurons does not lead to altered behavior after a difficult trial. Percentage of correct responses and the correct response latency in seconds is presented in trials following trials with a specific delay (5, 7.5, or 12.5 s) or cue duration (1, 0.5, 0.2 s). Sessions in which animals received saline injections are compared to sessions where they received CNO 5 mg/kg (CNO5) or CNO 10 mg/kg (CNO10). Data are presented as mean ± SD. Interactions between thalamus (MDL/MDM/eYFP) and striatum (DMS/VMS/eYFP) groups are tested with a three-way mixed repeated measures ANOVAs with dose and delay or cue duration as within-subject factors and group as between-subject factor. MDL *n* = 11, MDM *n* = 11, eYFP (thalamus) *n* = 13, DMS *n* = 10, VMS *n* = 12, eYFP (striatum) *n* = 13 rats. Download Table 1-1, DOCX file.

10.1523/ENEURO.0254-22.2022.f1-1 Extended Data Figure 1-1 Experimental design and behavioral task. ***A***, Schematic of the different experiments and groups of animals used. ***B***, Schematic of the behavioral task, showing the task stages of training and testing sessions. Download Figure 1-1, TIF file.

## Synthesis

Reviewing Editor: Mark Laubach, American University Decisions are customarily a result of the Reviewing Editor and the peer reviewers coming together and discussing their recommendations until a consensus is reached. When revisions are invited, a fact-based synthesis statement explaining their decision and outlining what is needed to prepare a revision will be listed below. The following reviewer(s) agreed to reveal their identity: NONE. Note: If this manuscript was transferred from JNeurosci and a decision was made to accept the manuscript without peer review, a brief statement to this effect will instead be what is listed below.

Your revised manuscript was reviewed by one of the original reviewers and a new reviewer. Your revisions satisfied the original reviewer. The new reviewer asked for three minor issues to be addressed. Please see the comments below and revise your paper accordingly. Your revised paper will be handled by editorial review only. The first two revisions were viewed as essential and either adding video data or addressing the lack of video and its implications for interpretations based on attention would also improve your paper.

(1) The task design was not clear. The use of CombiCages will not be common knowledge for many readers. A depiction of the arena used for self-motivated testing should be included in the manuscript itself. Move panel C from the supplemental figure to a new first figure, include a picture of the actual device, and preview the various designs in this figure to prepare the reader for the figures to come.

(2) Regarding statistics, why was ANOVA and normality testing done and then followed by either a Wilcoxon rank-sum test or t-test? Spearman rank correlation was also used. It seems that you have gone with normal based statistics but mixed in non-parametrics? Why? The rationale for this approach should be given. Some effects are surprisingly significant (for example, Figure 2B, left plot and others) and perhaps this is due to violation of assumptions of ANOVA for these data, especially response latencies, which are rarely normally distributed. The ANOVAs should be re-done with resampling for the F statistics and for all post-hoc tests. Also, mean +/‐ SEM with a large sample of animals tends to show very small bars, as in most plots in the paper. What do the line plots in Figure 2D and 2E look like if 95% confidence bands are instead used?

(3) Attention is discussed throughout the paper. No evidence that the animals attended to the stimuli is presented, other than manipulating stimulus delay and duration. Were video recordings made during the test sessions? If so, is there any evidence for the animals attending to the visual stimuli? Would this help explain why “slower animals omit more trials in the task”? Were omission errors due to non-task behaviors? These issues could be addressed with pose estimation methods (DeepLabCut or SLEAP, for example) or even simple center of mass tracking, provided that the animals’ orientations can be assessed. If no video is available for the present study, then the authors should really consider doing a small-scale study using video methods to support their stated interpretations, and the current manuscript should have text added to emphasize this major limitation of the study.
