# Supplementary material for: Prefrontal Cortical to Mediodorsal Thalamus Projection Neurons Regulate Posterror Adaptive Control of Behavior
Source: eNeuro. 2022 Nov 2;9(6):ENEURO.0254-22.2022. doi: 10.1523/ENEURO.0254-22.2022 (PMC9636992; doi:10.1523/ENEURO.0254-22.2022)
Supplement: Extended Data Table 1-1 — Inhibition of mPFC projection neurons does not lead to altered behavior after a difficult trial. Percentage of correct responses and the correct response latency in seconds is presented in trials following trials with a specific delay (5, 7.5, or 12.5 s) or cue duration (1, 0.5, 0.2 s). Sessions in which animals received saline injections are compared to sessions where they received CNO 5 mg/kg (CNO5) or CNO 10 mg/kg (CNO10). Data are presented as mean ± SD. Interactions between thalamus (MDL/MDM/eYFP) and striatum (DMS/VMS/eYFP) groups are tested with a three-way mixed repeated measures ANOVAs with dose and delay or cue duration as within-subject factors and group as between-subject factor. MDL n = 11, MDM n = 11, eYFP (thalamus) n = 13, DMS n = 10, VMS n = 12, eYFP (striatum) n = 13 rats. Download Table 1-1, DOCX file. [file enu-eN-NWR-0254-22-s01.docx]

| **Parameter** | **Trial type t-1** | **Condition** | **MDL** | **MDM** | **eYFP (thalamus)** | **DMS** | **VMS** | **eYFP (striatum)** |
| --- | --- | --- | --- | --- | --- | --- | --- | --- |
| **% Correct on trial t**  variable delay | **5 s** | Saline | 53.14 ± 8.72 | 57.21 ± 9.48 | 67.65 ± 12.01 | 71.09 ± 7.50 | 66.88 ± 9.36 | 69.51 ± 10.02 |
|  |  | CNO5 | 57.72 ± 8.83 | 52.34 ± 8.38 | 63.55 ± 10.62 | 66.48 ± 7.67 | 62.00 ± 13.02 | 67.73 ± 10.44 |
|  |  | CNO10 | 58.73 ± 15.24 | 52.79 ± 10.86 | 66.88 ± 11.96 | 64.71 ± 4.70 | 62.18 ± 11.07 | 69.33 ± 11.13 |
|  | **7.5 s** | Saline | 51.03 ± 10.24 | 53.28 ± 10.84 | 65.49 ± 10.91 | 65.60 ± 6.40 | 61.92 ± 8.20 | 65.90 ± 11.14 |
|  |  | CNO5 | 56.46 ± 10.11 | 48.78 ± 8.66 | 61.87 ± 10.98 | 65.11 ± 8.46 | 59.34 ± 15.01 | 65.59 ± 12.20 |
|  |  | CNO10 | 53.67 ± 12.04 | 48.45 ± 7.72 | 62.12 ± 11.11 | 63.37 ± 7.17 | 61.27 ± 10.61 | 66.41 ± 8.89 |
|  | **12.5 s** | Saline | 65.07 ± 11.78 | 60.51 ± 12.24 | 72.25 ± 8.34 | 73.42 ± 2.92 | 66.70 ± 11.92 | 75.23 ± 9.64 |
|  |  | CNO5 | 66.72 ± 13.71 | 53.27 ± 14.82 | 71.97 ± 8.34 | 72.80 ± 4.37 | 67.55 ± 10.73 | 72.51 ± 9.08 |
|  |  | CNO10 | 61.70 ± 15.02 | 55.24 ± 13.07 | 72.53 ± 10.27 | 72.60 ± 4.95 | 68.99 ± 11.69 | 75.13 ± 6.26 |
| **Correct response latency (s)** **on trial t**  variable delay | **5 s** | Saline | 1 ± 0.19 | 1.13 ± 0.32 | 0.78 ± 0.19 | 1.20 ± 0.16 | 1.23 ± 0.31 | 1.08 ± 0.18 |
|  |  | CNO5 | 1 ± 0.17 | 1.07 ± 0.26 | 0.78 ± 0.16 | 1.13 ± 0.17 | 1.18 ± 0.25 | 1.06 ± 0.16 |
|  |  | CNO10 | 1.05 ± 0.22 | 1.14 ± 0.28 | 0.77 ± 0.18 | 1.15 ± 0.15 | 1.20 ± 0.27 | 1.02 ± 0.16 |
|  | **7.5 s** | Saline | 1.08 ± 0.17 | 1.17 ± 0.30 | 0.84 ± 0.23 | 1.26 ± 0.15 | 1.32 ± 0.35 | 1.15 ± 0.17 |
|  |  | CNO5 | 1.08 ± 0.16 | 1.17 ± 0.21 | 0.85 ± 0.22 | 1.21 ± 0.17 | 1.26 ± 0.27 | 1.13 ± 0.19 |
|  |  | CNO10 | 1.13 ± 0.23 | 1.15 ± 0.25 | 0.83 ± 0.21 | 1.19 ± 0.12 | 1.26 ± 0.27 | 1.09 ± 0.17 |
|  | **12.5 s** | Saline | 1.01 ± 0.17 | 1.17 ± 0.35 | 0.79 ± 0.18 | 1.21 ± 0.14 | 1.25 ± 0.34 | 1.11 ± 0.16 |
|  |  | CNO5 | 1.03 ± 0.18 | 1.14 ± 0.34 | 0.81 ± 0.20 | 1.19 ± 0.20 | 1.21 ± 0.24 | 1.10 ± 0.17 |
|  |  | CNO10 | 1.09 ± 0.18 | 1.19 ± 0.35 | 0.82 ± 0.21 | 1.16 ± 0.11 | 1.21 ± 0.28 | 1.08 ± 0.17 |
| **% Correct on trial t**  variable cue duration | **1 s** | Saline | 47.25 ± 10.09 | 41.66 ± 10.18 | 60.23 ± 7.78 | 49.93 ± 5.98 | 52.39 ± 8.09 | 62.78 ± 7.05 |
|  |  | CNO5 | 42.43 ± 11.59 | 41.39 ± 10.73 | 58.28 ± 6.76 | 50.33 ± 8.77 | 50.62 ± 10.63 | 65.19 ± 6.74 |
|  |  | CNO10 | 38.74 ± 14.24 | 39.54 ± 13.72 | 59.43 ± 5.74 | 49.42 ± 5.23 | 48.45 ± 10.88 | 62.76 ± 6.96 |
|  | **0.5 s** | Saline | 45.11 ± 12.15 | 38.93 9.49 | 56.73 ± 4.73 | 51.23 ± 4.44 | 49.94 ± 8.00 | 61.40 ± 7.93 |
|  |  | CNO5 | 40.49 ± 11.21 | 40.98 ± 8.10 | 54.57 ± 8.10 | 52.70 ± 6.70 | 49.85 ± 10.32 | 61.71 ± 6..39 |
|  |  | CNO10 | 41.30 ± 14.22 | 36.12 ± 10.80 | 56.15 ± 7.92 | 50.09 ± 6.52 | 46.44 ± 8.95 | 61.86 ± 7.31 |
|  | **0.2 s** | Saline | 56.16 ± 11.78 | 47.73 ± 12.03 | 68.72 ± 9.15 | 60.33 ± 6.25 | 60.84 ± 9.36 | 72.62 ± 8.14 |
|  |  | CNO5 | 50.44 ± 14.31 | 47.19 ± 11.14 | 67.20 ± 10.90 | 59.44 ± 6.61 | 61.21 ± 12.29 | 69.77 ± 6.58 |
|  |  | CNO10 | 45.19 ± 13.74 | 42.43 ± 16.54 | 67.22 ± 10.87 | 60.15 ± 3.66 | 58.51 ± 10.91 | 71.31 ± 7.03 |
| **Correct response latency (s) on trial t**  variable cue duration | **1 s** | Saline | 0.91 ± 0.23 | 0.96 ± 0.28 | 0.65 ± 0.15 | 1.10 ± 0.14 | 1.06 ± 0.27 | 0.91 ± 0.13 |
|  |  | CNO5 | 0.95 ± 0.20 | 0.91 ± 0.25 | 0.66 ± 0.15 | 1.04 ± 0.15 | 1.03 ± 0.27 | 0.92 ± 0.11 |
|  |  | CNO10 | 0.97 ± 0.17 | 0.95 ± 0.32 | 0.65 ± 0.15 | 1.05 ± 0.16 | 1.01 ± 0.24 | 0.95 ± 0.09 |
|  | **0.5 s** | Saline | 1.02 ± 0.22 | 1.14 ± 0.30 | 0.79 ± 0.16 | 1.26 ± 0.13 | 1.23 ± 0.25 | 1.06 ± 0.16 |
|  |  | CNO5 | 1.09 ± 0.22 | 1.05 ± 0.26 | 0.80 ± 0.20 | 1.18 ± 0.14 | 1.20 ± 0.28 | 1.05 ± 0.09 |
|  |  | CNO10 | 1.09 ± 0.26 | 1.18 ± 0.69 | 0.80 ± 0.19 | 1.18 ± 0.15 | 1.17 ± 0.25 | 1.11 ± 0.10 |
|  | **0.2 s** | Saline | 0.95 ± 0.20 | 1.09 ± 0.33 | 0.72 ± 0.16 | 1.24 ± 0.14 | 1.18 ± 0.30 | 1.05 ± 0.15 |
|  |  | CNO5 | 0.99 ± 0.20 | 1.01 ± 0.25 | 0.73 ± 0.19 | 1.20 ± 0.17 | 1.18 ± 0.32 | 1.05 ± 0.14 |
|  |  | CNO10 | 1.03 ± 0.28 | 1.03 ± 0.33 | 0.72 ± 0.20 | 1.21 ± 0.17 | 1.16 ± 0.30 | 1.05 ± 0.12 |

**Supplementary Table 1 Inhibtion of mPFC projection neurons does not lead to altered behavior after a difficult trial.** Percentage of correct responses and the correct response latency in seconds is presented in trials following trials with a specific delay (5, 7.5 or 12.5 s) or cue duration (1, 0.5, 0.2 s). Sessions in which animals received saline injections are compared to sessions where they received CNO 5 mg/kg (CNO5) or CNO 10 mg/kg (CNO10). Data are presented as mean ± SD. Interactions between thalamus (MDL/ MDM/ eYFP) and striatum (DMS/ VMS/ eYFP) groups are tested. MDL n = 11, MDM n = 11, eYFP (thalamus) n = 13, DMS n = 10, VMS n= 12, eYFP (striatum) n = 13 rats.
