# Supplementary figures and images for: Prefrontal Cortical to Mediodorsal Thalamus Projection Neurons Regulate Posterror Adaptive Control of Behavior
Source: eNeuro. 2022 Nov 2;9(6):ENEURO.0254-22.2022. doi: 10.1523/ENEURO.0254-22.2022 (PMC9636992; doi:10.1523/ENEURO.0254-22.2022)

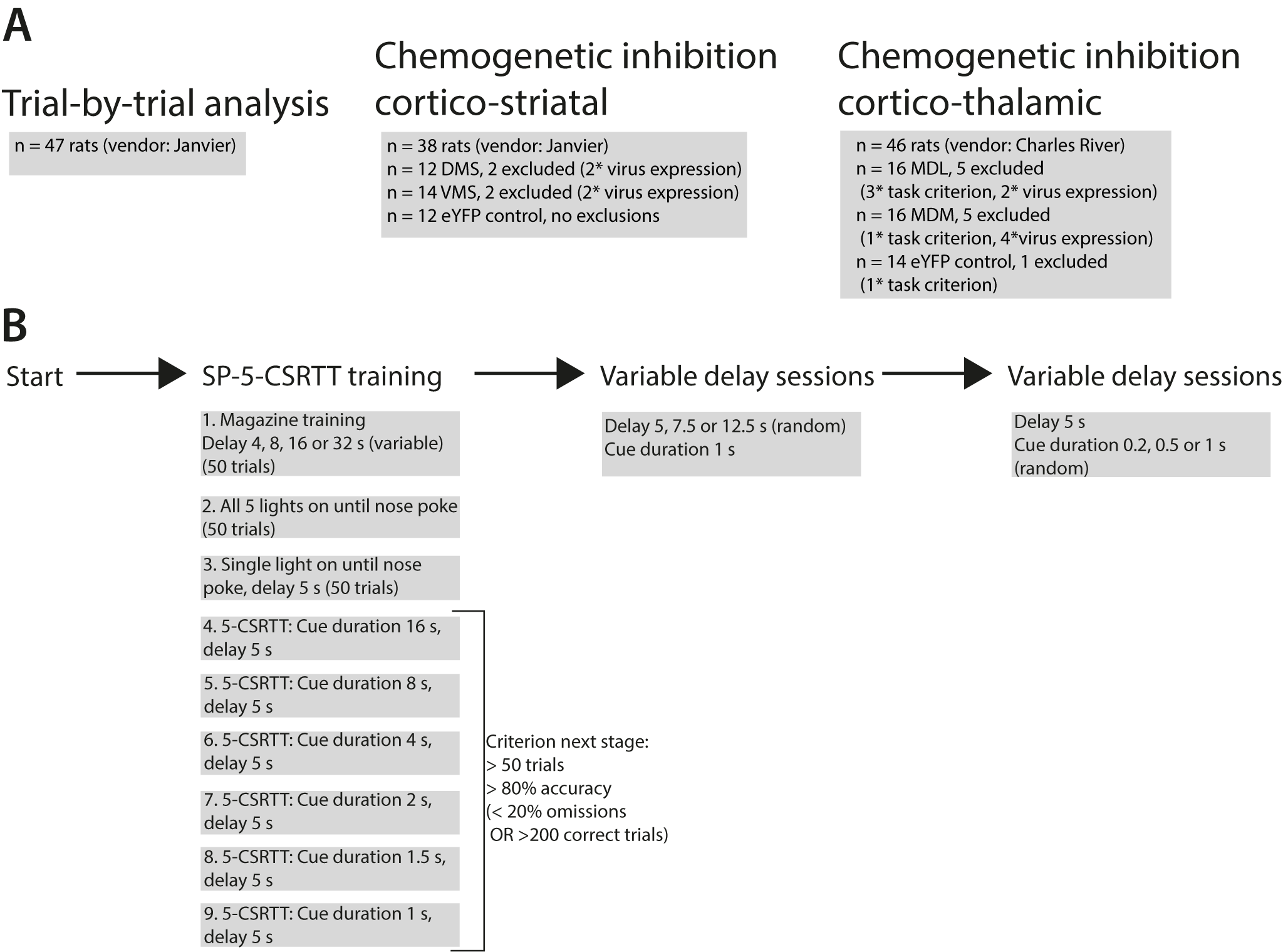

Supplement: Extended Data Figure 1-1 — Experimental design and behavioral task. A, Schematic of the different experiments and groups of animals used. B, Schematic of the behavioral task, showing the task stages of training and testing sessions. Download Figure 1-1, TIF file. [file enu-eN-NWR-0254-22-s02.tif]
